# Vitamin D and methylarginines in chronic kidney disease (CKD)

**DOI:** 10.1371/journal.pone.0185449

**Published:** 2017-10-04

**Authors:** Claudia Torino, Patrizia Pizzini, Sebastiano Cutrupi, Rocco Tripepi, Giovanni Tripepi, Francesca Mallamaci, Carmine Zoccali

**Affiliations:** CNR-IFC, Clinical Epidemiology and Physiopathology of Renal Diseases and Hypertension & Nephrology and Renal Transplantation Unit, Reggio Calabria, Italy; University of Milan, ITALY

## Abstract

**Background:**

Vitamin D associates with the plasma concentration of the endogenous inhibitor of the nitric oxide system asymmetric dimethyl arginine (ADMA) and cross-sectional studies in CKD patients treated with the vitamin D receptor activator paricalcitol show that plasma ADMA is substantially less than in those not receiving this drug.

**Methods:**

In the frame of a randomized, double-blind, placebo controlled trial, the Paracalcitol and ENdothelial fuNction in chronic kidneY disease (PENNY), we investigated whether vitamin D receptor activation by paricalcitol (2 μg/day x 12 weeks) affects the plasma concentration of ADMA and symmetric dimethyl arginine (SDMA) in 88 patients with stage 3 to 4 CKD.

**Results:**

Paricalcitol produced the expected small rise in serum calcium and phosphate and a marked PTH suppression. However, ADMA [Paricalcitol: baseline 0.75 μMol/L (95%CI: 0.70–0.81), 12 week 0.72 μMol/L (95%CI: 0.66–0.78); Placebo: baseline 0.75 μMol/L (95%CI: 0.70–0.90) 12 weeks 0.70 μMol/L (95%CI: 0.66–0.74)] and SDMA [Paricalcitol: baseline 0.91 μMol/L (95%CI: 0.82–1.00), 12 week 0.94 μMol/L (95%CI: 0.82–0.1.06); Placebo: baseline 0.91 μMol/L (95%CI: 0.82–1.06) 12 weeks 0.99 μMol/L (95%CI: 0.88–1.10)] remained unchanged during the trial and 2 weeks after stopping these treatments.

**Conclusions:**

Paricalcitol does not modify plasma ADMA and SDMA in patients with stage 3–4 CKD. The apparent beneficial effects of paricalcitol on ADMA registered in cross-sectional studies is likely attributable to confounding by indication rather than to a true effect of this drug on ADMA metabolism.

## Introduction

Asymmetric dimethyl arginine (ADMA) and its symmetric enantiomer, symmetric dimethyl arginine (SDMA), are endogenous inhibitors of the enzyme that regulates the synthesis of nitric oxide (NO), NO synthase. ADMA and SDMA have been associated with atherosclerosis and with cardiovascular[1] and renal diseases[2]. ADMA has been implicated in the pathogenesis of various conditions including Alzheimer's dementia[3], insulin resistance [4], thyroid dysfunction[5], fertility [6] and erectile dysfunction[7]. These compounds accumulate in chronic kidney disease and are considered as full-fledged uremic toxins[8].

NO synthase is expressed in bone cells and in these cells NO functions as a transducer of mechanosensitive signals [9]. In the rat plasma ADMA is inversely related with bone mass [10] and in mice this methylarginine inhibits osteoblastic differentiation, an effect which is reversed by the administration of the NO precursor L-arginine[11]. Sparse observations in various conditions support the hypothesis that ADMA may be a relevant player in mineral bone disorders. Indeed, in type 2 diabetics the plasma concentration of this biomarker associates with serum PTH independently of other factors [12]. In the general population [13] and in the elderly population [14] low 25-hydroxyvitamin D3 (25-OH Vit D) correlates inversely with ADMA and seasonal fluctuations in 25-OH Vit D associate with reciprocal ADMA fluctuations [13]. An inverse relationship between ADMA and 25-OH Vit D has been described in stage G5D CKD patients [15]. The possibility that the link between vitamin D in CKD and ADMA is causal in nature is suggested by the fact that in cross sectional analyses the plasma concentration of ADMA is halved in patients on treatment with vitamin D receptor antagonists like paricalcitol as compared to patients not receiving such a treatment [16]. Of note, in the same study an apparent dose-response relationship between the dose of paricalcitol and the degree of ADMA suppression was described [16]. However precious for hypothesis generation, cross sectional analyses are inherently inadequate to assess causation[17] and therefore it remains unresolved whether activation of the vitamin D receptor may suppress ADMA. We have now tested the nature of the link between vitamin D and methylarginines within the frame of a recent double-blind, randomized, controlled clinical trial, the Paracalcitol and ENdothelial fuNction in chronic kidneY disease (PENNY) study (clinicaltrials.gov identifier: NCT01680198). In this trial we systematically measured ADMA and SDMA in each study participant at all time-points of the study with no missing sample and herein report the results of analyses related to these methylarginines.

## Materials and methods

The study protocol was approved by the ethics committee of our Institution, Comitato Etico Sezione Sud—Regione Calabria, and a written informed consent was obtained from each participant.

### Patients

The protocol of the PENNY trial and the CONSORT flow diagram of this trial are reported into detail in the source study[18]. Briefly, PENNY is a double-blind, randomized, parallel group trial (ClinicalTrials.gov identifier, NCT01680198) enrolling 88 patients with CKD stage 3 to 4, age ranging between 18 and 80 years, parathormone ≥ 65 pg/ml, serum total Ca between 2.2 and 2.5 mmol/L and phosphate levels between 2.9 mg/dL and 4,5 mg/dL, not in treatment with vitamin D compounds or anti-epileptic drugs, without neoplasia or symptomatic cardiovascular disease or liver disease. Patients who met the inclusion criteria were randomized (1:1) to receive 2 μg paricalcitol once daily or matching placebo for 12 weeks after a 2-week run-in. The dose of paricalcitol was adjusted on the basis of serum parathormone and Ca and the maximum dose allowed was 2 μg daily. No vitamin D compounds were allowed during the trial. Demographic, clinical and biochemical data of the two study arms are listed in Table 1.

**Table 1:** Demographic, clinical and biochemical characteristics of the two study arms at baseline.

	Active group (n = 44)	Placebo group (n = 44)	P
Age (years)	63±11	62±12	0.65
Male sex (%)	59%	70%	0.27
Current smokers (%)	12%	19%	0.37
Past smokers (%)	45%	41%	0.66
Diabetes (%)	34%	36%	0.82
BMI (kg/m^2^)	29±5	29±5	0.66
Systolic/Diastolic BP (mmHg)	123±16/73±9	129±21/73±11	0.16/0.81
Heart rate (beats/min)	67±8	68±10	0.64
Cholesterol (mg/dL)	164±41	162±43	0.84
HDL Cholesterol (mg/dL)	47±11	50±13	0.18
LDL Cholesterol (mg/dL)	88±34	88±36	0.91
eGFR_Cyst_ (ml/min/1.73m^2^)	34±12	29±13	0.06
Hemoglobin (g/dL)	12±2	12±2	0.49
Calcium (mmol/L)	2.25±0.12	2.21±0.10	0.16
Phosphate (mmol/L)	1.20±0.19	1.23±0.16	0.29
Parathormone (pg/mL)	102 (81–146)	102 (85–154)	0.70
FGF-23 (pg/mL)	64.7 (52.7–81.2)	78.0 (53.7–103.1)	0.07
1,25-OH vitamin D (pmol/L)	101.4±41.6	93.6±41.8	0.32
25-OH vitamin D (nmol/L)	33±16	38±16	0.19
C reactive protein (mg/L)	1.18 (0.68–3.02)	2.49 (0.99–3.74)	0.11
ADMA (μMol/L)	0.75±0.19	0.75±0.16	0.82
SDMA (μMol/L)	0.91±0.30	0.91±0.30	0.92

Data are expressed as mean± SD, median and inter-quartile range or as percent frequency as appropriate.

Abbreviations: BMI, body mass index; BP, blood pressure; LDL, low-density lipoprotein; HDL, high-density lipoprotein; GFR, glomerular filtration rate; FGF-23, fibroblast growth factor-23.

### Laboratory measurements

Serum calcium, phosphate, glucose, lipids were measured in the routine clinical pathology laboratory at our Institution. Serum creatinine was measured by the Roche enzymatic, IDMS calibrated method and serum cystatin C by the Siemens Dade Behring kit and the GFR was calculated by the CKD-Epi creatinine-cystatin formula [19]. Plasma parathormone was measured by immunoradiometric assay (DiaSorin Stillwater, MN, USA), 25 OH Vit D and 1,25 (OH)_2_ Vit D by radioimmunoassay (Immunodiagnostic Systems, Boldon, UK). Plasma ADMA [20] and SDMA [21] were measured by validated ELISA methods by using commercially available kits by DLD Gesellschaft für Diagnostika, Hamburg, Germany. Normal ranges of plasma ADMA and SDMA determined by this method are 0.40–0.75 and 0.30–0.70 mMol/L, respectively. Plasma samples were kept frozen at -80° degrees, without freeze-thaw cycles, until analysis and ADMA and SDMA measurements were measured in a single assay.

### Statistical analysis

Data are reported as mean ± standard deviation (normally distributed data), median and inter-quartile range (non-normally distributed data) or as percent frequency, and comparison between groups were made by independent T-Test, Mann-Whitney Test, or Chi Square test. Correlates of methylarginines and of paricalcitol-induced changes in methylarginines were analyzed by using the Pearson’s correlation coefficient (on log_10_ transformed data, when appropriate). All comparison were appropriately adjusted for multiple testing. The effect of paricalcitol on plasma ADMA and SDMA was analyzed by comparing the changes in these indices in paricalcitol-treated and untreated patients by using the T-Test for independent observations. The potential effect modification by demographic (age and gender) and bone mineral disorder biomarkers at baseline [calcium, phosphate, 25-OH Vit D, 1,25-(OH)_2_ Vit D, PTH and FGF23] on the relationship between allocation arm and plasma methylarginines was investigated by standard interaction analyses by creating appropriate multiplicative terms [22]. All P values in this analyses were appropriately adjusted for multiple comparisons (Bonferroni correction). Differences in risk factors at baseline not controlled by randomization and due to chance were accounted by using multiple regression. Data analysis was performed by SPSS for Windows (version 20.0, Chicago, Illinois, USA).

## Results

At baseline, patients randomized to paricalcitol and placebo did not differ for demographic, clinical and biochemical characteristics, except for the eGFR which tended to be higher in patients receiving paricalcitol (P = 0.06) (Table 1). Vitamin D insufficiency (>25 nmol/L <75 nmol/L) or deficiency (<25 nmol/L) was present in the vast majority (95%) of patients with no between-arms difference (42 patients in each group). The proportion of patients with elevated ADMA and SDMA (i.e. presenting levels above the upper limit of the normal range of each indicator) was 39% for ADMA and 73% for SDMA. Alongside with strictly comparable plasma levels of bone disorder biomarkers, the average values of these biomarkers at baseline were very similar in the two study arms (Table 1). As reported in detail in the source study[18] and in Table 2, drug treatments, including ACE inhibitors, sartans, hypoglicemizing agents, statins and proton pump inhibitors, were similar between the two groups except for calcium carbonate, this latter compound being more frequently administered in patients on placebo (22.7%) than in those in the paricalcitol arm (0%) (P = .003).

**Table 2:** Drug treatments in the two study arms.

	Active group (n = 44)	Placebo group (n = 44)	P
ACE inhibitors (%)	47.7%	47.7%	1.00
Angiotensin Receptor Blockers (%)	59.1%	47.7%	0.29
Diuretics (%)	34.1%	47.7%	0.19
Alpha and Beta Blockers (%)	36.4%	38.6%	0.83
Calcium Antagonists (%)	40.9%	52.3%	0.29
Statins (%)	45.5%	52.5%	0.52
Omega 3 polyunsaturated fatty acids (%)	27.3%	11.4%	0.06
Hypoglycemizing agents (%)	6.8%	18.2	0.11
Insulin (%)	13.6%	15.9%	0.76
Antiplatelet agents (%)	50.0%	54.5%	0.83
Nitrates (%)	9.1%	4.5%	0.40
Erythropoietin stimulating agents (%)	2.3%	2.0%	0.56
Iron (%)	11.4%	13.6%	0.75
Calcium carbonate (%)	0.0%	22.7%	0.003
Proton pump inhibitors (%)	63.6%	50.0%	0.20

Data are expressed as percent frequency and compared by the Chi Square Test (with the continuity correction when appropriate).

### Functional correlates of ADMA and SDMA in CKD patients

In the cross sectional analysis made at baseline, ADMA and SDMA were inter-related (r = 0.272, P<0.01). ADMA and SDMA correlated inversely with the GFR-Cys-creat and SDMA exhibited a stronger link with this parameter (r = -0.566, P<0.001) than ADMA (r = -0.376, P<0.001). Furthermore, SDMA but not ADMA was inversely related with age (r = -0.275, P = 0.009), serum phosphorus (r = 0.293, P = 0.006), PTH (r = 0.306, P = 0.004), 1.25-(OH)_2_ Vit D (r = -0.243, P = 0.0023) as well as with FGF23 (r = 0.226, P = 0.034). However, in analyses adjusting for age and the GFR-cys-creat, two relevant confounders for the link between SDMA and bone mineral disorder biomarkers, all these associations failed to maintain an independent association with SDMA [Phosphorus-SDMA β = 0.001, P = 0.99; PTH-SDMA β = 0.178, P = 0.06; 1,25-(OH)_2_ Vit D-SDMA β = -0.055, P = 0.54; FGF23-SDMA β = 0.135, P = 0.11].

### Effects of paricalcitol on plasma ADMA and SDMA

**Fig 1:**
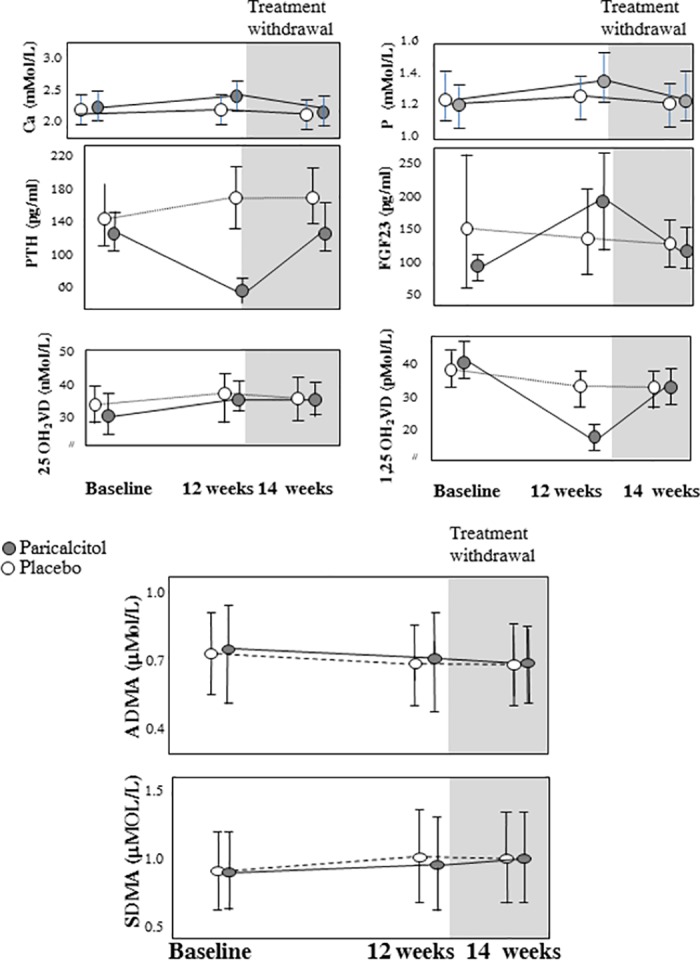
Changes in bone mineral disorder biomarkers, ADMA and SDMA according to the treatment group (paricalcitol/placebo). The bars correspond to 95%CI.

Paricalcitol produced a modest rise in serum calcium and phosphate, the expected suppression in PTH and 1,25-OH vitamin D, a marked rise in FGF23 and no change in 25-OH vit D (see Fig 1 and Ref [18]). However, vitamin D receptor activation by this drug largely failed to modify ADMA and SDMA that remained unchanged both during paricalcitol and placebo and after stopping these treatments (Fig 1). These results did not change after adjustment for the eGFR (P = 0.97 and P = 0.43) or for calcium carbonate treatment, i.e. the two variables that were different at baseline in two study arms. No effect modification by age, gender, baseline 25-OH vitamin D, 1,25-OH vitamin D, calcium, phosphate, PTH and FGF23 on ADMA, SDMA was noted.

## Discussion

In this trial vitamin D receptor activation by paricalcitol produced the expected effects on calcium, phosphate, PTH, FGF23 and 1,25-(OH)_2_ vitamin D but left completely unmodified the plasma concentration of ADMA and SDMA.

Even though there is still no trial based on clinical end-points targeting ADMA and SDMA, these methylarginines are regarded as important risk factors for death [23] [24], cardiovascular disease [25] and progression to kidney failure[26] [27]. Various drugs can modify the plasma concentration of ADMA. ACE-inhibitors like perindopril[28] or enalapril[29] or zofenopril[30] as well as the angiotensin II receptor blocker eprosartan[29] produce a modest lowering in the plasma concentration of ADMA but it is unclear whether this effect is related to their anti-hypertensive action or to interference with ADMA metabolism. Metformin reduces ADMA in diabetics [31] and the peroxisome proliferator activated receptor agonist rosiglitazone lowers ADMA in patients with insulin resistance[32]. Estrogens in postmenopausal woman have a consistent but minor ADMA lowering effect [33]. Overall, these drugs have modest effects on ADMA and given the interference of ADMA and SDMA with a pathway (NO synthesis) of critical relevance for cardiovascular[1] and renal disease[2], the search of new drugs producing a biologically relevant decrease of these methylarginines is an important undertaking with potential implications for clinical practice. Vascular dysfunction appears to be tightly associated with high ADMA levels in CKD patients[34]. Paricalcitol coherently improves vascular dysfunction by increasing the NO-dependent forearm flow mediated vasodilatation in response to ischemia[18] [35] in these patients. Also because the previously mentioned links of ADMA with a fundamental biomarker of mineral bone disorders like PTH [12] and the inverse seasonal fluctuations of ADMA and 25-OH Vit D[36], vitamin D receptor activation appears to be an interesting, novel pathway to explore to mitigate ADMA accumulation in CKD. Descriptive, cross-sectional studies would support the possibility of a biologically important effect of vitamin D on ADMA levels in CKD because the plasma concentration of this compound is more than halved in patients on chronic paricalcitol treatment as compared to those that do not take this drug[16]. Furthermore, categorical analyses in the same study show that among patients in the fourth ADMA quartile the proportion of patients not on paricalcitol is 3.5 times lower than that of patients in the first ADMA quartile[16].

The PENNY study contemplated the creation of a biological databank for exploring scientific hypotheses related with vitamin D activation in CKD and serum samples were available for all patients enrolled into this trial. Therefore this double blind randomized trial represents an ideal basis for experimentally testing whether this drug can modify plasma methylarginines levels.

Like in a previous study applying an inactive form of vitamin D [37], in the present study paricalcitol substantially reduced circulating and perhaps intracellular 1,25 –OH vitamin D levels thereby limiting the VDR exposure to the main endogenous agonist of this receptor. Our data show that a powerful synthetic VDR agonist given at adequate pharmacological doses sufficient to suppress PTH (a major effect of VDR stimulation) largely fails to modify ADMA levels. Overall it seems unlikely that low intracellular 1,25-vitamin D might justify the complete lack of the ADMA-response to VDR stimulation.

In contrast with the cross-sectional analysis by Oliva-Damaso et al.,[16] paricalcitol had a robustly null effect on the plasma concentration of ADMA and SDMA and extensive interaction analyses showed no modification of the effect of paricalcitol on these methylargines in relationship with demographic factors (age, gender) and the main biomarkers of the mineral bone disorder in CKD including calcium, phosphate, 25-OH vitamin D, 1,25-(OH)_2_ vitamin D, PTH and FGF23. Confounding by indication is a well-known bias of associations detected in cross-sectional studies[38]. The possibility that paracalcitol was preferentially prescribed to patients at lower risk for cardiovascular disease, i.e. to patients who typically exhibit lower ADMA levels, might explain the much lower levels in the plasma concentration of this compound in patients treated with this drug as compared to patients not receiving this treatment. Even though Oliva-Damaso et al., made meaningful adjustments to minimize confounding by indication, this bias is difficult to remove in observational studies and therefore the possibility of residual confounding in the same study appears much likely. A survival advantage by paricalcitol was noted in observational studies analyzed by state of art statistical techniques including propensity matching [39] and marginal structural models [40] while no signal for a reduced death risk or an attenuation in the progression of left ventricular hypertrophy was registered in a subsequent double blind randomized trial testing the effects of paricalcitol in predialysis CKD patients[41]. However intriguing, the hypothesis that an ADMA lowering effect by paricalcitol may mediate the apparent survival advantage of patients treated by this drug is negated by the robustly negative findings in the PENNY trial.

In conclusion, analyses performed within the frame of a double blind randomized trial, do not confirm cross-sectional analyses showing a favorable effect of this drug on circulating levels of the two main methylargines, ADMA and SDMA. The purported favorable effect of paracalcitol on cardiovascular outcomes does not appear justified by an ADMA and/or SDMA lowering effect of this vitamin D receptor activator.
